# The Complete Mitochondrial Genome and Bio-Ecological Notes of *Lhasella potala* Zhou, Da, Zahradník & Bai, 2025 (Coleoptera: Ptinidae: Anobiinae), a Pest Infesting Wooden Structural Components

**DOI:** 10.3390/insects17060549

**Published:** 2026-05-25

**Authors:** Xuan Zhou, Zhuoma Pubu, Rongrong Shen, Wa Da, Petr Zahradník, Ming Bai

**Affiliations:** 1 Dawa Innovation Studio, Institute of Plateau Biology of Xizang Autonomous Region, Lhasa 850000, China; z_h_y_xuan@163.com (X.Z.); sws_pz@163.com (Z.P.); 2 State Key Laboratory of Animal Biodiversity Conservation and Integrated Pest Management, Institute of Zoology, Chinese Academy of Sciences, Beijing 100101, China; shenrongrong@ioz.ac.cn; 3 Forestry and Game Management Research Institute, Strnady 136, CZ-252 02 Jíloviště, Czech Republic; zahradnik@vulhm.cz; 4 Hebei Key Laboratory of Animal Diversity, College of Life Science, Langfang Normal University, Langfang 065000, China; 5 Academy of Plateau Science and Sustainability, Qinghai Normal University, Xining 810016, China

**Keywords:** anobiids, cultural heritage, mitogenome, storage beetles, wooden structure

## Abstract

*Lhasella potala* Zhou, Da, Zahradník & Bai, 2025, a wood-boring pest, infests wooden components of the Potala Palace and threatens structural integrity. To support conservation-oriented pest management, we sequenced and characterized its complete mitochondrial genome and conducted comparative and phylogenetic analyses within Ptinidae. The mitogenome is 15,399 bp long and contains 37 genes (13 PCGs, 22 tRNAs, two rRNAs, and a control region). It exhibits high A + T content (74.23%), positive AT-skew (0.16), and negative GC-skew (−0.26). All PCGs employed ATT, ATA, ATG, ATC, or TTG as the start codons and TAA, TAG, or single T as the stop codons. Comparisons with *Lasioderma serricorne* (Fabricius, 1775), *Stegobium paniceum* (Linnaeus, 1758), and *Gastrallus laevigatus* (Olivier, 1790) revealed similar genome sizes and conserved gene order. Ka/Ks analysis showed that *cox1* evolves most slowly, whereas *nad4* evolves fastest. Nucleotide diversity was low (Pi = 0.18–0.34). Phylogenetic analysis supports a close relationship between *G. laevigatus* and *L. potala*. Field observations indicate one generation per year, with peak activity from May to July. These results provide a basis for evolutionary studies and conservation of historic wooden architecture.

## Simple Summary

Wood-boring beetles can cause serious damage to historic wooden buildings. *Lhasella potala*, has been found infesting the Potala Palace in China, a UNESCO World Heritage Site. To better understand this species, we analyzed its complete mitochondrial genome and compared it with related species. The genome shows typical features of beetles and a stable gene arrangement. Our results indicate that this species is closely related to *Gastrallus laevigatus* and has a relatively slow rate of genetic change in some genes. Field observations show that it produces one generation per year, with peak activity from May to July. These findings improve our understanding of the evolution and bio-ecology of Ptinidae and provide baseline information for future studies on anobiids associated with wooden cultural heritage.

## 1. Introduction

The family Ptinidae Latreille, 1802 currently comprises 12 subfamilies and nearly 4200 described species worldwide, representing a morphologically and ecologically diverse group of beetles [1,2]. Among them, anobiids (historically treated as a separate family Anobiidae) have been demonstrated to form a monophyletic lineage based on molecular phylogenetic evidence [3]. Consequently, their placement within the family Ptinidae has gained broad acceptance in recent systematic treatments [4,5,6]. Many members of anobiids are characterized by their wood-boring habits, with larvae often exploiting a wide range of plant-derived substrates [7]. Notably, several anobiids are recognized as economically important pests of stored products and wooden materials. Well-known examples include the furniture beetle *Anobium punctatum* (DeGeer, 1774), the library beetle *Nicobium castaneum* (Olivier, 1790), and the book-boring beetle *Falsogastrallus sauteri* Pic, 1914. These species are widely distributed across different climatic regions and can cause severe damage to furniture, books, archival documents, medicinal products, and various commodities of animal and plant origin [8,9,10,11], leading to considerable economic losses and management costs.

Beyond their impact on stored products, some anobiids pose an increasing threat to historical wooden structures and cultural heritage sites. *Lhasella potala* is a typical member and was recorded infesting wooden structural components of the Potala Palace. Its larvae bore into and degrade the wood, potentially compromising the structural integrity of ancient buildings and posing a tangible risk to the conservation of wooden cultural heritage [12]. Indeed, evidence of its harmful activity was recorded as early as July 1988 during restoration work on the palace, when construction personnel explicitly noted that the insect posed a substantial threat to the wooden structures [13].

Mitochondrial genomes are widely used as molecular markers in phylogenetic and evolutionary studies due to their conserved gene content, relatively stable gene order, maternal inheritance, and rapid evolutionary rates. Compared with single-locus markers, complete mitochondrial genome data provide higher phylogenetic resolution and more robust support for evolutionary inference at multiple taxonomic levels [14,15]. With the increasing availability of mitogenomic data, their application has expanded beyond phylogenetic reconstruction to practical pest management. In particular, mitochondrial genomes provide reliable species-specific targets for the development of diagnostic markers, facilitating the rapid and accurate identification of morphologically similar species or early-stage invasive populations, especially in quarantine inspection and monitoring programs [16,17]. However, complete mitochondrial genomes specifically sequenced for Ptinidae remain scarce in public databases. Prior to this study, only four identified species of Ptinidae (including three anobiids: *Stegobium paniceum*, *Gastrallus laevigatus*, and *Lasioderma serricorne*) had reported mitogenomes available in NCBI (see Table 1). Notably, the assembly for *Gastrallus laevigatus* was incomplete, although it contained all 13 PCGs and two rRNAs.

The aims of this study were to sequence and characterize the complete mitochondrial genome of *L. potala* for the first time to compare its mitochondrial genome features with those of other available anobiids and to preliminarily assess its phylogenetic position within Ptinidae. To achieve these objectives, comprehensive analyses were conducted on genome structure, nucleotide composition, codon usage, and molecular evolutionary rates, with comparative assessments against three other anobiids. Furthermore, based on year-round field observations, we summarized key biological and ecological characteristics of this species. Together, these results provide useful molecular and bio-ecological data for future studies on the evolution and systematics of Ptinidae, while also contributing baseline information for further research on anobiids associated with wooden cultural heritage.

## 2. Materials and Methods

### 2.1. Specimen Collection, Identification, and Preservation

A total of 20 adult specimens of *L. potala* were collected from the wooden structures of the Potala Palace in Lhasa, Xizang Autonomous Region, China, in June 2024. The specimens were placed in Eppendorf tubes, flash frozen in liquid nitrogen, and stored at –80 °C for subsequent use.

### 2.2. DNA Extraction and Sequencing

Total genomic DNA was extracted from whole specimens using the TIANamp Genomic DNA Kit (Tiangen, Beijing, China), following the manufacturer’s protocol. The integrity of the extracted DNA was assessed using agarose gel electrophoresis (DYCP-31DN, Beijing Liuyi Biotechnology Co., Ltd., Beijing, China), and DNA purity was evaluated by measuring absorbance values with a spectrophotometer (NanoDrop 2000c, Thermo Fisher Scientific, Waltham, MA, USA). Qualified DNA samples were sent to Nanjing Genscript Biotech Co., Ltd. (Nanjing, China) for Illumina DNA library preparation and paired-end DNA sequencing. The Illumina sequencing library, with an insert size of 350 bp, was constructed using the NEBNext^®^ Ultra^TM^ II DNA Library Prep Kit for Illumina^®^ (New England Biolabs, Ipswich, MA, USA) and sequenced on the Illumina Novaseq 6000 platform (Illumina, San Diego, CA, USA), producing a total of 6 Gb of data.

### 2.3. Sequence Assembly, Annotation, and Analyses

Mitochondrial genome assembly and the annotation of PCGs and rRNAs were performed using Geneious prime 2025 (version 2025.0) [21]. The published mitochondrial genome of *Lasioderma serricorne* was used as the reference sequence for reference-guided assembly. The start and stop codon positions of the PCGs were manually inspected and adjusted as necessary. The newly sequenced mitochondrial genome of *L. potala* was deposited in GenBank.

Annotation of the tRNAs was conducted using the MITOS2 web server (http://mitos.bioinf.uni-leipzig.de/, accessed on 20 March 2025) [22]. The secondary structure models of the tRNAs were predicted using the tRNAscan-SE 2.0 web server (http://lowelab.ucsc.edu/tRNAscan-SE/, accessed on 20 March 2025), and visualized with Adobe Illustrator 2024 (version 28.8.0). The complete mitochondrial genome map was generated using OGDRAW (https://chlorobox.mpimp-golm.mpg.de/OGDraw.html, accessed on 25 March 2025) [23]. The base composition and relative synonymous codon usage (RSCU) of the complete mitochondrial genome were calculated using MEGA 11 (version 11.0) [24]. The RSCU figure was generated using the R packages ggplot2 (version 3.5.1) and aplot (version 0.2.3) in R (version 4.4.1). The AT-skew and GC-skew were calculated using the formulas: AT-skew = (A − T)/(A + T) and GC-skew = (G − C)/(G + C), following the method of Perna and Kocher [25].

### 2.4. Comparative Mitogenomic Analysis of Four Anobiids

Orthologous PCGs were identified from all pairwise species comparisons. Each orthologous gene pair was aligned using MAFFT (version 7.427) [26] with the default settings to preserve codon positions. The resulting alignments were used to estimate nonsynonymous (Ka) and synonymous (Ks) substitution rates with KaKs_Calculator (version 2.0) [27] under the MLWL model. Then, the Ka/Ks ratio was calculated to evaluate selective pressure on each PCG. To evaluate nucleotide-level genetic variation, orthologous sequences of the 13 PCGs and two rRNAs from all taxa were globally aligned using MAFFT (version 7.427) with the “–auto” option. Nucleotide diversity (Pi) for each gene was calculated based on these alignments using DnaSP (version 6.12.03) [28]. For structural comparisons, complete mitochondrial genome sequences were aligned with Mauve (version 2.4.0) [29] under default parameters to identify locally collinear blocks (LCBs) and to detect potential genomic rearrangements such as inversions and translocations.

### 2.5. Phylogenetic Analysis

In addition to the newly sequenced mitochondrial genome of *L. potala*, four additional mitogenomes from Ptinidae were included in the phylogenetic analysis (Table 1). To root the phylogenetic tree, two species from Dermestidae (*Dermestes lardarius*, NC053876.1; *D. maculatus*, MG457037.1), a family within the same superfamily Bostrichoidea as Ptinidae, were selected as outgroups. PhyloSuite (version 1.2.3) was used to extract gene sequences from GenBank files and import the extraction results into MAFFT (version 7.427) [26] for multiple sequence alignment. Then, MACSE (version 2.07) [30] was used to optimize PCG alignments. Gblocks (version 0.91b) [31] was used to trim the alignments of nucleotide sequences of PCGs. The nucleotide sequences of 13 PCGs and two rRNAs were concatenated using PhyloSuite to generate a combined dataset for subsequent phylogenetic analyses. ModelFinder [32] was used to select the optimal partitioning strategy and evolutionary models for concatenated datasets. Bayesian inference (BI) in MrBayes (version 3.2.7) [33] and Maximum Likelihood (ML) in IQ-TREE (version 3.0.1) [34] were conducted using plugins in PhyloSuite. Finally, phylogenetic trees were visualized and annotated using iTOL (https://itol.embl.de/, accessed on 28 March 2025) [35].

### 2.6. Bio-Ecological Observations

Since April 2025, bio-ecological observations of insect infestation in wooden structural components were conducted within the Potala Palace. Passive monitoring devices, including yellow sticky traps and small flight-interception traps, were deployed at each monitoring site, with a total of 28 monitoring points established. Samples were collected and recorded at weekly intervals at all sites. Based on the collected specimens and the in situ observational data obtained during the sampling process, the biological and ecological characteristics of the species were systematically compiled and analyzed.

## 3. Results

### 3.1. Structural Features and Gene Orders in L. potala Mitochondrial Genome

**Figure 1:**
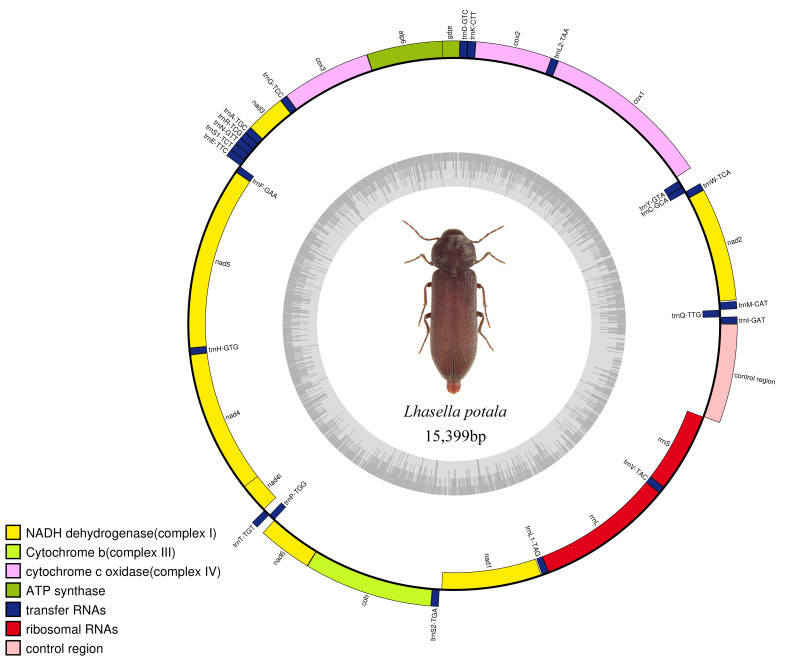
A circular map of the mitochondrial genome of *L. potala*. The habitus image of the insect is reproduced from [12], reproduced with permission from the copyright holder, © Magnolia Press.

The complete mitochondrial genome of *L. potala* (Accession No. PV298246.1) is 15,399 bp and comprises 37 genes, including 13 protein-coding genes (PCGs: *atp6*, *atp8*, *cob*, *cox1*, *cox2*, *cox3*, *nad1*, *nad2*, *nad3*, *nad4*, *nad4l*, *nad5*, and *nad6*), two ribosomal RNA genes (*rrnL* and *rrnS*), and 22 transfer RNA (tRNA) genes, namely *trnA-TGC*, *trnC-GCA*, *trnD-GTC*, *trnE-TTC*, *trnF-GAA*, *trnG-TCC*, *trnH-GTG*, *trnI-GAT*, *trnK-CTT*, *trnL1-TAG*, *trnL2-TAA*, *trnM-CAT*, *trnN-GTT*, *trnP-TGG*, *trnQ-TTG*, *trnR-TCG*, *trnS1-TCT*, *trnS2-TGA*, *trnT-TGT*, *trnV-TAC*, *trnW-TCA*, and *trnY-GTA*. Additionally, a control region was identified in the non-coding portion of the genome (Figure 1). Among these genes, 4 PCGs, 2 rRNAs, and 8 tRNAs (*nad1*, *nad4*, *nad4l*, *nad5*, *rrnL*, *rrnS*, *trnF-GAA*, *trnH-GTG*, *trnP-TGG*, *trnL1-TAG*, *trnV-TAC*, *trnQ-TTG*, *trnC-GCA*, *trnY-GTA*) are located on the minority (N) strand, whereas the remaining 9 PCGs and 14 tRNAs are encoded on the majority (J) strand (Table 2). Moreover, the genome has a compact organization with a total of 18 gene junctions involving either intergenic spacers or overlapping regions. The cumulative length of intergenic spacers is 80 bp, and that of overlapping regions is 33 bp. The largest intergenic spacer (34 bp) occurs between *trnY* and *cox1*, followed by a 17 bp spacer between *trnS2* and *nad1*. The most extensive overlap (−8 bp) is found between *trnW* and *trnC* (Table 2).

The nucleotide composition of the complete mitochondrial genome exhibits a strong A + T bias, with the proportions of A, T, C, and G being 43.09%, 31.14%, 16.25%, and 9.51%, respectively, resulting in the A + T content of 74.23% and the G + C content of 25.77%. The PCG region is 11,018 bp in length and also shows a high A + T content (71.9%) and relatively low GC content (28.1%), with low AT-skew (−0.108) and GC-skew (−0.037) values. The tRNA region is 1438 bp in length and exhibits an even higher A + T content (76.91%) and a near-zero AT-skew (0.02), indicating a relatively conserved and balanced composition. The rRNA region is 2021 bp in length and characterized by a high T content (46.12%) and a negative AT-skew (−0.171) value, reflecting a predominance of thymine over adenine, as well as a relatively low G + C content. The control region (D-loop) is 875 bp and displays an extremely high A + T content (88.23%), which suggests the presence of regulatory or repetitive sequences, a common feature observed in the mitochondrial genomes of many insect species [36,37] (Table 3).

**Figure 2:**
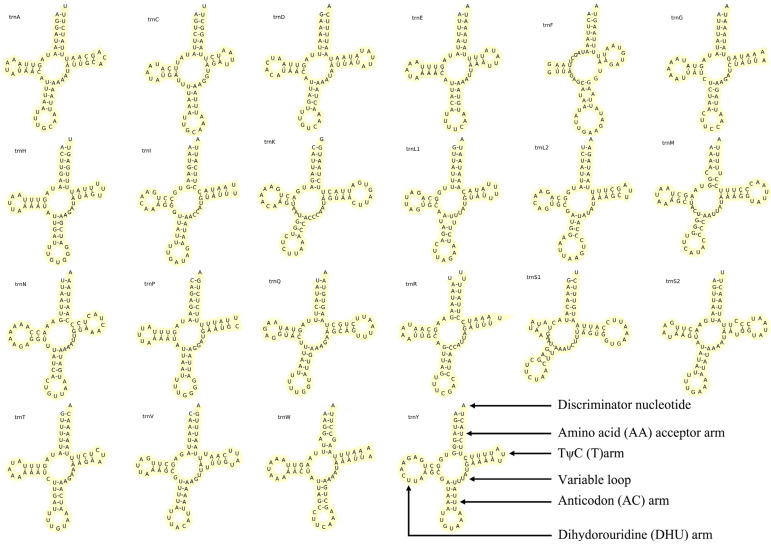
The predicted secondary structures of tRNAs in the complete mitochondrial genome of *L. potala*. The 22 tRNA genes were marked with abbreviations in the top left.

The lengths of the 22 tRNAs range from 62 to 71 bp. All tRNAs are predicted to fold into the canonical cloverleaf secondary structure, with well-formed acceptor stems, TΨC arms, anticodon arms, and DHU arms (Figure 2). We detected 23 mismatched base pairs, including one AA mismatch in *trnF*, and 22 GU mismatches in other tRNAs.

### 3.2. PCGs and Codon Usage

**Figure 3:**
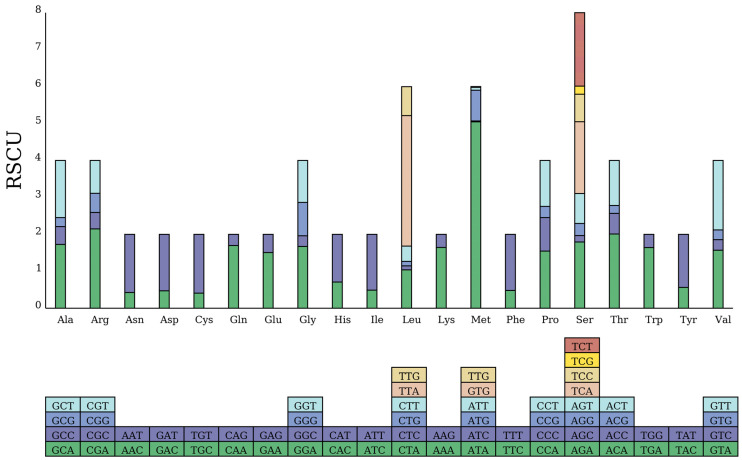
Relative synonymous codon usage of *L. potala*. For methionine (Met), the codon AUA was overwhelmingly preferred, resulting in a disproportionately large contribution in the stacked bar plot. Other synonymous codons occurred at very low frequencies and are therefore difficult to distinguish visually.

The RSCU values of the PCGs in the mitochondrial genome of *L. potala* are shown in Figure 3. Among the stop codons, UAA is overwhelmingly favored (RSCU = 1.85), while UAG is rarely used (RSCU = 0.15). According to the invertebrate mitochondrial genetic code, UGA is interpreted as tryptophan (Trp, RSCU = 1.64) rather than as a stop codon, representing a typical mitochondrial codon reassignment pattern widely reported in insects [38,39]. Similarly, AUA is interpreted as methionine (Met, RSCU = 5.05) instead of isoleucine, whereas AGA and AGG are interpreted as serine (Ser, RSCU = 1.79 and 0.33, respectively) rather than arginine. Moreover, the codon usage pattern shows a marked bias toward A/U-ending codons, consistent with the overall AT-rich nature of the genome. Codons such as UUU (Phe, RSCU = 1.52), AUU (Ile, RSCU = 1.51), and UUA (Leu, RSCU = 3.53) are highly preferred, whereas G/C-ending synonymous codons like GCC (Ala, RSCU = 0.48) and CGC (Arg, RSCU = 0.44) are significantly underrepresented. In addition, codon usage for certain amino acids exhibits strong specificity. For example, more than 70% of leucine residues are encoded by UUA (RSCU = 3.53), and AUA accounts for approximately 85% of methionine codons (RSCU = 5.05).

### 3.3. Comparative Analysis of Mitochondrial Genomes Among Four Anobiids

#### 3.3.1. Mitogenomic Synteny Analysis

**Figure 4:**
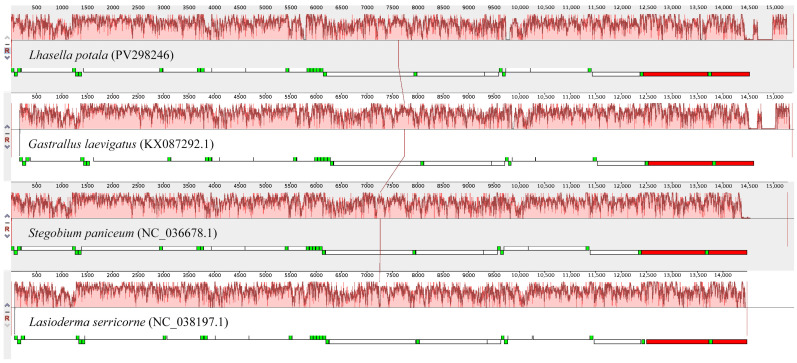
Progressive Mauve analyses showing the genome size variations and global rearrangement structures of four anobiids mitochondrial genomes. Each genome is displayed horizontally, and homologous regions shared among the mitochondrial genomes are shown as colored locally collinear blocks. Red connecting lines indicate syntenic relationships among corresponding regions, while the red similarity profiles represent sequence conservation across the aligned mitochondrial genomes.

A whole-genome alignment of the mitochondrial genomes of *L. potala*, *Lasioderma serricorne*, *Stegobium paniceum*, and *Gastrallus laevigatus* was performed to assess structural conservation across these four anobiids. The analysis revealed that all four mitochondrial genomes share a similar size and exhibit high synteny, with consistent gene order and orientation. No structural rearrangements, including gene inversions or translocations, were detected, indicating that the mitochondrial genome architecture has remained highly conserved during the evolution of these species, without significant genomic reorganization (Figure 4).

#### 3.3.2. Selective Pressure on Mitochondrial Core PCGs

**Figure 5:**
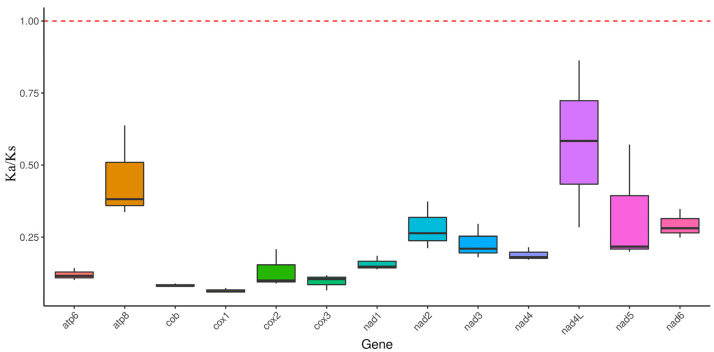
The Ka/Ks ratios of 13 PCGs among four anobiids. The red dashed line indicates the neutral selection threshold (Ka/Ks = 1).

The ratio of nonsynonymous to synonymous substitution rates (Ka/Ks) is an important indicator for understanding the evolutionary dynamics of protein-coding sequences [40,41]. Comparative analyses of mitochondrial genomes were conducted among four anobiids. Ka/Ks values were calculated for the 13 PCGs (Figure 5). The results revealed that the Ka/Ks ratios for all 13 PCGs across the four anobiid species were less than 1, indicating predominant purifying selection. Among these genes, *cox1* exhibited the lowest Ka/Ks ratio while *nad4L* displayed the highest.

#### 3.3.3. Analysis of Mitochondrial Nucleotide Diversity (Pi)

**Figure 6:**
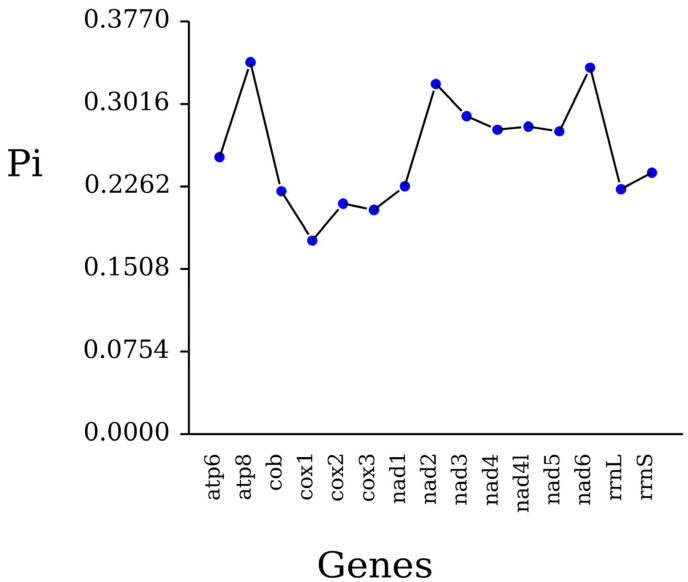
The mitochondrial nucleotide diversity of 13 PCGs and two rRNAs among four anobiids.

Nucleotide diversity analysis was performed on 13 PCGs and two rRNAs from four anobiids using DnaSP (version 6.12.03). The results indicated relatively low overall fluctuation in Pi values (range: 0.18–0.34), with a mean of 0.26. This suggests that nucleotide diversity across the selected gene regions is generally conserved among these species, with limited variation. Further analysis revealed that the *cox1* gene was the most conserved, exhibiting the lowest Pi value (0.18), which reflects its relatively low nucleotide diversity across the examined species. In contrast, the *atp8* and *nad6* genes displayed comparatively higher nucleotide diversity, with Pi values of 0.34 and 0.33, respectively (Figure 6).

### 3.4. Phylogenetic Relationships Inferred from Mitochondrial Genomes 

**Figure 7:**
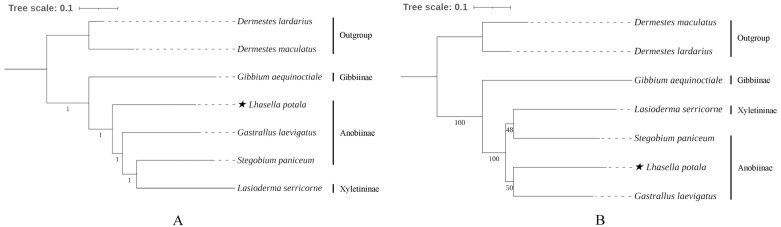
Phylogenetic trees reconstructed based on the concatenated nucleotide dataset of 13 PCGs and two rRNAs using BI (**A**) and ML (**B**). Numbers above branches indicate posterior probabilities (PP) in (**A**) and bootstrap support values (BS) in (**B**). The focal species studied in this paper are indicated by stars.

Phylogenetic trees were reconstructed using both BI and ML methods based on the concatenated nucleotide alignment of the 13 PCGs and two rRNAs (Figure 7). While all internal nodes within the family Ptinidae were strongly supported in the BI analysis (posterior probability = 1.0), the anobiids nodes in the ML tree received consistently low bootstrap support (≤50%). This marked discrepancy suggests that the phylogenetic signal in the current mitochondrial dataset is insufficient to robustly resolve the relationships among these ptinid species. Based on the available mitochondrial data, it is tentatively suggested that *Gastrallus laevigatus* is more closely related to *L. potala* (the focal species of this study) than to the other species analyzed.

### 3.5. Bio-Ecological Notes

**Figure 8:**
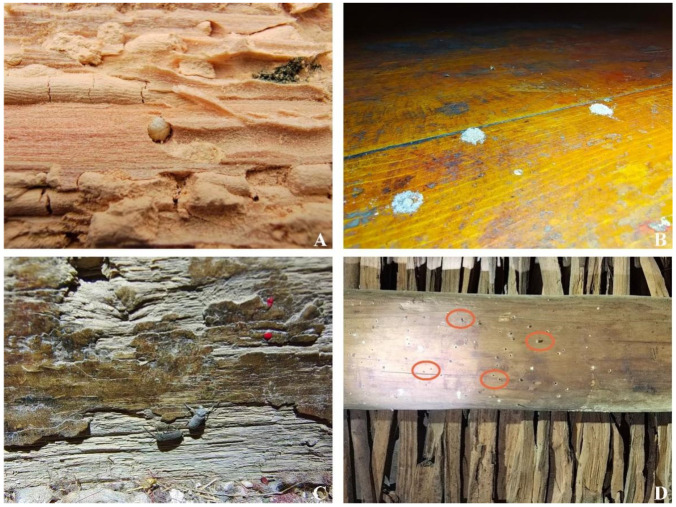
Habitat and biological characteristics of *L. potala*. (**A**) Larva and larval galleries within wooden structural components (from [12], reproduced with permission from the copyright holder, ©Magnolia Press). (**B**). Frass accumulated at the gallery opening. (**C**) Adult active near wooden structural components. (**D**) Dead adult blocking the gallery opening (indicated by red circles).

The species is univoltine, completing one generation per year. Larvae remain entirely within wooden structural components throughout their development (Figure 8A), where they feed on wood tissues and produce conspicuous frass accumulations at the gallery openings (Figure 8B). The diameter of the larval galleries is approximately 3 mm. Around May, larvae pupate within the wood and subsequently emerge as adults. Newly emerged adults are commonly observed moving on or around the surfaces of the wooden structures (Figure 8C). Adult females are generally larger than males. Adults feed little or not at all and exhibit limited flight capability. Mating typically occurs outside the galleries, after which oviposition takes place at the gallery entrances. By approximately July, adults begin to die off gradually. In addition, most field observations revealed a distinct plugging behavior at the gallery openings (Figure 8D).

## 4. Discussion

### 4.1. Mitochondrial Genome Structure

The complete mitochondrial genome of *L. potala* is 15,399 bp in length and contains the full complement of 37 genes typically present in insect mitochondrial genomes, including 13 PCGs, two rRNAs, and 22 tRNAs. The genome exhibits a highly compact organization and conserved gene order, conforming to the canonical structural features of insect mitochondrial genomes [38]. The overall base composition is characterized by a notable A + T bias, with an A + T content of 74.23%. This pronounced A + T enrichment represents a common feature of arthropod mitochondrial genomes, widely attributed to the combined effects of long-term mutational pressure and selective evolutionary constraints [14,42]. Beyond a mere structural trait, this compositional bias may confer adaptive advantages by influencing key mitochondrial processes such as transcription, replication efficiency, and energy metabolism. Consequently, it likely represents an important factor shaping mitogenomic evolution in *L. potala*.

### 4.2. Mitochondrial Codon Usage

The non-standard genetic code, where UGA codes for tryptophan, and AT-rich codon usage bias observed in this study represent hallmark features of arthropod mitochondrial genomes, which are widely conserved across diverse lineages including other insects [14,43]. This convergent evolutionary pattern reflects functional coincidental. Evidence indicates that the streamlining and optimization of the mitochondrial genetic system, including codon reassignment, reflect adaptations to its unique translational environment and energy metabolism requirements [44]. More broadly, as the central genetic component of the cellular energy factory, the evolution of the mitochondrial genome is often shaped by natural selection to align with organism-specific physiological states and external environmental pressures [45]. Therefore, the codon usage pattern of *L. potala* can be interpreted as part of a series of fine-tuned evolutionary adjustments in its mitochondrion, optimized for functional efficiency and adaptation to its specific ecological niche over long-term evolution.

### 4.3. Comparative Analysis of Mitochondrial Genomes

In comparison with other anobiid species, the mitochondrial genome architecture of *L. potala* was found to be highly conserved, with no evidence of substantial gene rearrangements or inversions. This structural conservation indicates a relatively stable evolutionary trajectory of the mitochondrial genome in this species. In the analysis of selective pressure on mitochondrial core PCGs, *cox1* displayed the lowest Ka/Ks ratio, suggesting it is the most conserved gene and has the slowest evolutionary rate within anobiids, whereas *nad4L* showed the highest evolutionary rate under purifying selection. Nucleotide diversity (Pi) analyses further revealed that core mitochondrial genes of *L. potala* exhibit generally low Pi values across the four anobiid species examined, suggesting a high degree of interspecific conservation in these regions. Notably, *cox1* exhibited the lowest Pi value, reflecting its slow evolutionary rate within anobiids. This result aligns with the well-established consensus that *cox1* is the most evolutionarily conserved mitochondrial protein-coding gene [46]. In contrast, the relatively higher Pi values observed for *atp8* and *nad6* indicate increased evolutionary variability in these loci, which may be associated with species-specific ecological adaptations [45].

### 4.4. Phylogenetic Implications for Ptinidae Systematics

Based on taxonomic priority, the family Anobiidae is currently treated as part of Ptinidae [2]. Although its position in the superfamily Bostrichoidea is well-established [20,47], the internal phylogenetic relationships within the family are still not well resolved, especially due to a lack of molecular phylogenetic studies. This study was limited by a small sample size and inadequate reference data for anobiid molecular systematics. Further research should include more samples and broader taxonomic representation, combining molecular and morphological evidence to provide a stronger molecular foundation for Ptinidae systematics.

### 4.5. Biological Characteristics

Parental care is observed in many insect species. For instance, in *Nicrophorus* beetles, both males and females provide extensive parental care, with a major benefit of male assistance being to help defend the brood and the carcass from competitors [48]. In *Forficula auricularia*, the female guards against intruders and physically blocks the nest entrance, serving as an active biological barrier [49]. In the present study, sealing of bore holes was frequently observed. However, whether this behavior represents brood guarding remains unclear. Additionally, the sex of the guarding parent (female, male, or both) requires further investigation.

## 5. Conclusions

In summary, the first complete mitochondrial genome of *L. potala* provides new insights into its genomic features, evolutionary patterns, and phylogenetic position within Ptinidae. Together with field-based observations, these results provide baseline molecular and bio-ecological data for future studies on the systematics and evolution of Ptinidae.

## Figures and Tables

**Table 1:** Mitochondrial genomes of Ptinidae from NCBI. A representative entry is shown for species with multiple records.

Family	Subfamily	Species	Completeness	Accession No.	Length (bp)	Resource	Note
Ptinidae	Anobiinae	*Lhasella potala*	Complete	PV298246.1	15,399	This study	Historically treated as the separate family Anobiidae
		*Stegobium paniceum*	Complete	NC036678.1	15,271	[18]	
		*Gastrallus laevigatus*	Partial	KX087292.1	15,361	Unpublished	
	Xyletininae	*Lasioderma serricorne*	Complete	NC038197.1	14,476	[19]	
	Gibbiinae	*Gibbium aequinoctiale*	Complete	KY549398.1	15,361	[20]	---

**Table 2:** The positions and features of the genes in the *L. potala* mitochondrial genome.

Gene	Strand	Position (bp)	Length (bp)	Anticodon	Start Codon	Stop Codon	Intergenic Length
*trnI*	J	1–64	64	GAT	---	---	0
*trnQ*	N	62–130	69	TTG	---	---	−3
*trnM*	J	130–198	69	CAT	---	---	−1
*nad2*	J	208–1207	1000	---	ATT	T--	9
*trnW*	J	1208–1273	66	TCA	---	---	0
*trnC*	N	1266–1327	62	GCA	---	---	−8
*trnY*	N	1327–1393	67	GTA	---	---	−1
*cox1*	J	1428–2925	1498	---	ATT	T--	34
*trnL2*	J	2926–2989	64	TAA	---	---	0
*cox2*	J	2990–3662	673	---	ATA	T--	0
*trnK*	J	3663–3733	71	CTT	---	---	0
*trnD*	J	3733–3800	68	GTC	---	---	−1
*atp8*	J	3801–3956	156	---	ATA	TAA	0
*atp6*	J	3950–4618	669	---	ATG	TAA	−7
*cox3*	J	4618–5404	787	---	ATG	T--	−1
*trnG*	J	5405–5468	64	TCC	---	---	0
*nad3*	J	5469–5820	352	---	ATT	T--	0
*trnA*	J	5821–5884	64	TGC	---	---	0
*trnR*	J	5884–5946	63	TCG	---	---	−1
*trnN*	J	5945–6010	66	GTT	---	---	−2
*trnS1*	J	6011–6077	67	TCT	---	---	0
*trnE*	J	6078–6140	63	TTC	---	---	0
*trnF*	N	6144–6208	65	GAA	---	---	3
*nad5*	N	6209–7922	1714	---	ATT	T--	0
*trnH*	N	7923–7985	63	GTG	---	---	0
*nad4*	N	7986–9318	1333	---	ATG	T--	0
*nad4l*	N	9312–9593	282	---	ATG	TAA	−7
*trnT*	J	9602–9664	63	TGT	---	---	8
*trnP*	N	9665–9728	64	TGG	---	---	0
*nad6*	J	9731–10,213	483	---	ATC	TAA	2
*cob*	J	10,213–11,347	1135	---	ATG	T--	−1
*trnS2*	J	11,348–11,414	67	TGA	---	---	0
*nad1*	N	11,432–12,367	936	---	TTG	TAG	17
*trnL1*	N	12,375–12,437	63	TAG	---	---	7
*rrnL*	N	12,438–13,710	1273	---	---	---	0
*trnV*	N	13,711–13,776	66	TAC	---	---	0
*rrnS*	N	13,777–14,524	748	---	---	---	0
D-loop	J	14,525–15,399	875	---	---	---	0

**Table 3:** Nucleotide composition and strand bias of *L. potala*.

Gene	Size (bp)	A%	T%	G%	C%	A + T%	G + C%	AT-Skew	GC-Skew
Complete genome	15,399	43.09	31.14	9.51	16.26	74.23	25.77	0.16	−0.26
PCGs	11,018	32.06	39.84	13.52	14.58	71.90	28.10	−0.11	−0.04
tRNAs	1438	39.22	37.69	13.07	10.01	76.91	23.09	0.02	0.13
rRNAs	2021	32.66	46.12	13.81	7.42	78.77	21.23	−0.17	0.30
D-loop	875	44.23	44.00	4.46	7.31	88.23	11.77	0.003	−0.24

## Data Availability

The mitochondrial genome sequence generated in this study has been deposited in the NCBI GenBank database under accession number PV298246.1.
